# Cased-based education rounds—the eternal heart of an international training program

**DOI:** 10.3389/fped.2024.1306020

**Published:** 2024-02-23

**Authors:** Colm R. Breatnach, Alejandro Floh, Melanie Hamilton, Briseida Mema

**Affiliations:** ^1^ Department of Critical Care Medicine, The Hospital for Sick Children, Toronto, ON, Canada; ^2^ Department of Pediatrics, Faculty of Medicine, University of Toronto, Toronto, ON, Canada

**Keywords:** case-based rounds, psychological safety, teaching, pediatrics, case-based learning

## Abstract

Case-based teaching or “Morning Rounds” have been used in medical education for more than a century and remain a cornerstone for teaching in many training programs. Our Pediatric Critical Care Medicine (PCCM) program was established forty years ago and has retained this form of teaching since its inception. Case-based rounds have consistently had the highest evaluation of all curricula in our program. Here we review the history of how these rounds were introduced in medical education, provide data from the learners' evaluation of these case-based rounds, and discuss the strengths and potential drawbacks of this form of teaching from an educational theories perspective with the hope that they can be used by other Pediatric Critical Care training programs.

## Introduction

Our Pediatric Critical Care Medicine (PCCM) training program was established in 1981 and has trained approximately 450 pediatric intensivists from 50 different countries. As a program that trains this diverse a group, we aimed to review which one of our formal educational activities ranked most highly from our trainees in the past 10 years. Our program provides 12 formal educational curricula that target knowledge and skills essential to a Pediatric Intensivist. These include: (1) Case-Based Rounds (2) Academic Half Day—a weekly protected 3 h teaching session for trainees; (3) Extracorporeal Life Support curriculum, (4) Continuous Renal Replacement Curriculum, (5) PCCM Simulation Curriculum, (6) Medical Humanities Curriculum, (7) Visiting Professor Rounds, (8) Research Methods Curriculum, (9) Mechanical Ventilation Curriculum, (10) Morbidity and Mortality Rounds, (11) Discovery Rounds and (12) Inter Professional Practice Rounds. Feedback on each training cirricula is evaluated every three months by 25–30 PCCM subspeciality trainees (fellows) who participate in all of these curricula. We found that case-based rounds or so called “Morning Rounds” were consistently evaluated as the most effective and rewarding educational activity. Their longevity and perceived value warrant a reflection about their structure, educational benefits, and threats of case-based rounds from an educational perspective for the global critical care community.

### History of case-based rounds

Case-based rounds or “Morning rounds” were originally founded by William Osler at Johns Hopkins University for teaching and peer review (1). Osler reviewed the patients while role-modeling taking history, performing a clinical exam, teaching about the patient, but also asking questions. At Hopkins and other institutions, these rounds served for the Chief of Service to achieve the necessary oversight of the patient's care and for trainees to get access to their professors as they were planning management of the patients (2). In the decades that followed, case-based rounds evolved from its main focus being quality assurance to purely educational and a core element of training through case-based discussion (3). Despite subsequent modifications to their timing, format, and membership, patient-centered focus has remained at its core (4). Nevertheless, questions have been raised about potential negative impacts associated with the “pimping” or peppering of trainees with questions (5). Our PCCM program has maintained this unique forum as a safe space for staff and trainees to gather and discuss mortalities, debrief morally and ethically distressing situations, challenge clinical decision-making, and cover important educational topics.

### Structure of case-based rounds in our institution

**Figure 1:**
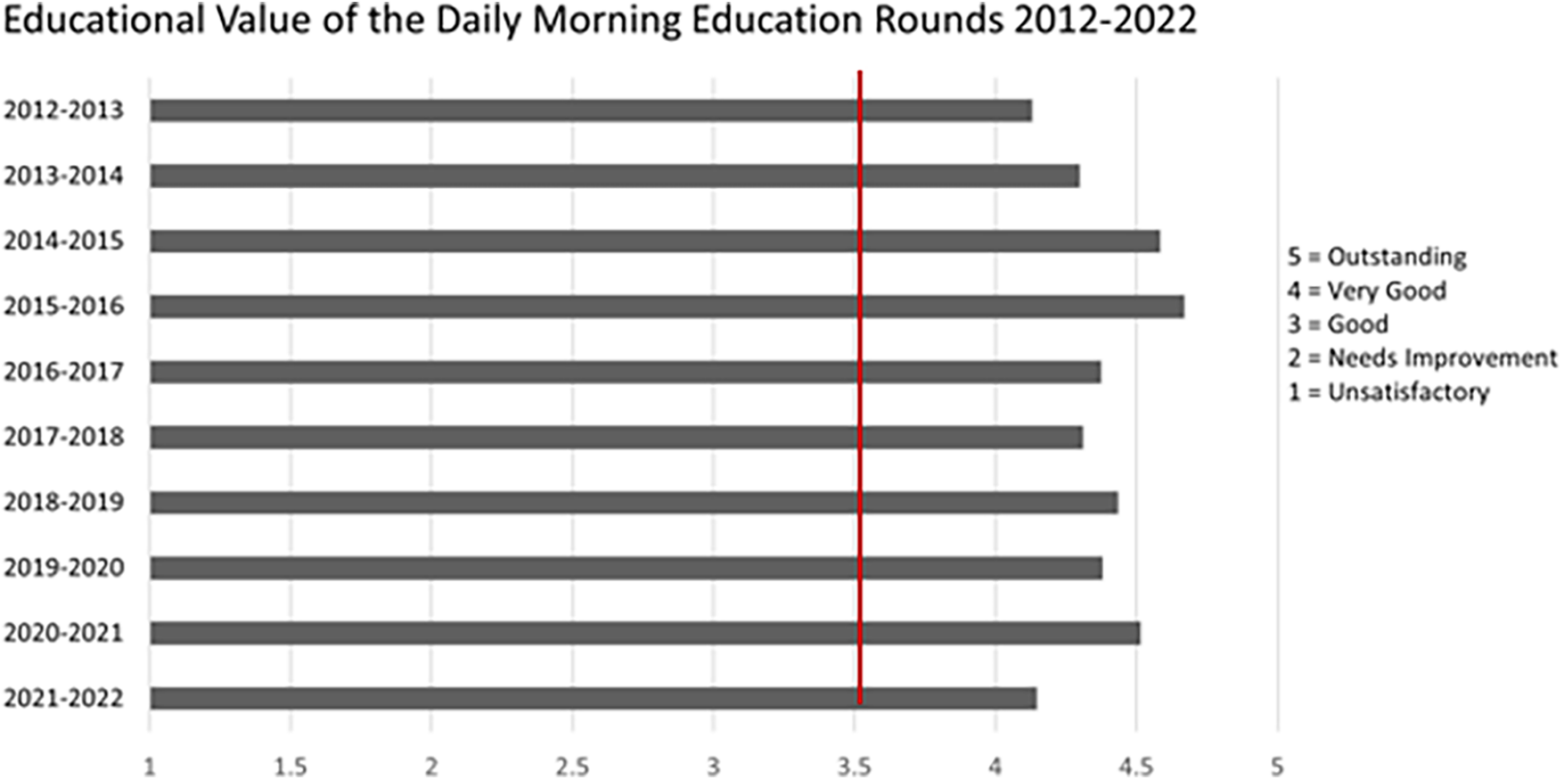
Anonymous evaluation of case-based rounds from 25 to 30 PCCM subspeciality trainees (fellows) yearly for the past 10 years. The numbers represent the means. Questions asked: “What is the quality of the following curricula provided in your training program?”. Survey scale (1 = Unsatisfactory, 2 = Needs Improvement, 3 = Good, 4 = Very Good, 5 = Outstanding). Red line presents the average evaluation of all the other eleven curricula in our program.

The rounds occur daily on weekdays for 60 min. They follow and are seen as an extension of the inter-professional clinical bedside rounds, with the focus now turned to the education of the multidisciplinary team. Patients discussed might be new admissions, but also patients who have been in the hospital longer, and infrequently patients who have been discharged but present opportunities for learning and discussion. While family members participate and contribute to the bedside clinical rounds, they do not participate in case-based rounds. The rounds are held in a conference room in proximity to the clinical area to make possible the team's timely return to the bedside should a clinical emergency arise. All trainees are expected to attend, but one remains responsible for emergencies in the clinical unit. The Respiratory Therapist (RT) in charge and the Registered Nurse (RN) in charge provide clinical care for the patients who are being discussed. This allows the bedside team (trainee, RN, RT) to attend the teaching pertinent to their patient. In addition, the RN and RT Educator and our Advance Nurse Specialists attend and co-teach with the faculty responsible for clinical service that week. Intermittently, faculty from other disciplines are invited to teach about a topic relevant to the patient being discussed. In the majority of the cases, our PCCM Morning Rounds are facilitated by the two faculty responsible for clinical service on the Pediatric and Cardiac Critical Care Units. The discussions focus on challenging clinical cases that address relevant patient care issues and provide a holistic overview of the challenges in managing complex cases. The lead faculty slowly unravel the details of the cases while questioning, prompting, and guiding trainees, receiving input from peers and faculty throughout. The sessions are attended by the majority of our faculty and trainees, bedside nurses and respiratory therapists, thereby enriching the discussions with their vast collective expertise. Discussions cover relevant topics that vary from how to navigate challenging conversations with families to resuscitation. The relevant literature is discussed, when available, to support decisions. The evaluations of the last ten years are included here (Figure 1).

### Benefits of case-based rounds

Morning rounds, although originating as a peer review forum, are also a case-based learning approach. Harvard Business School adopted this approach in 1920 across all their learning curricula stating: “…when students are presented with a case, they place themselves in the role of the decision maker and identify the problem they are faced with…” (6). A 2012 review found that case-based learning was superior to didactic lectures in student satisfaction, engagement, and motivation (7). More recent reports support the benefits of this approach in assessment and knowledge acquisition, invluding deeper conceptual understanding (8, 9), virtual learning for international collaborations (10) and social advocacy (11).

What are the benefits of this forum of teaching when we look at its important elements from an theoretical educational perspective? Foremost, the patient-focused or case-based teaching employed in this forum is strongly backed in the education literature supporting the notion that knowledge is optimized when acquired through solving real-world problems (12). Second, the infamous questioning, if done right, is a necessary process for the teacher to assess the foundation upon which they must build. Questioning is as important for the learner because the vast literature on self-assessment suggests that trainees benefit from being questioned, assessed, and forced to commit to an answer, as they may misjudge their level of understanding when receiving the answers during didactic teaching (13). Third, we can look at the benefits of combined trainee and faculty attendance and their contributions to the case with questions and answers, by incorporating two educational principles: guided discovery and co-construction of knowledge. Guided discovery learning combines pointing the way to understanding or problem-solving by a guide (in our case the faculty) with the discovery of facts, and solutions by trainees themselves, as they explore and discuss, while drawing upon their own experience and existing knowledge (14). Being questioned without fully knowing the solution introduces learners to the element of struggle. Internal struggle can be a potent mechanism for learning when guided by supervisors and advanced peers in the discovery of solutions (14). This guided discovery and the variety of cases contribute to a solid foundation of knowledge and preparation for future learning (14). Co-construction of knowledge is a collaborative model of learning where participants, in the case of our case-based rounds—faculty, trainee, RT and RN—embark on answering questions and building knowledge together (15). The benefits of co-learning are multiple, such as lessening power differentials, normalizing uncertainty, modeling lifelong learning, enriching learning with different perspectives and improving overall learning quality (16). Most importantly, by embarking on collaborative learning the team augments their collective competence (17). Lastly, exploring the difference of opinions and lenses through which problems are viewed introduces the learner to the complexity and nuances of cases that are inherent to the art and science of medicine (18).

### Potential risks of case-based rounds

**Figure 2:**
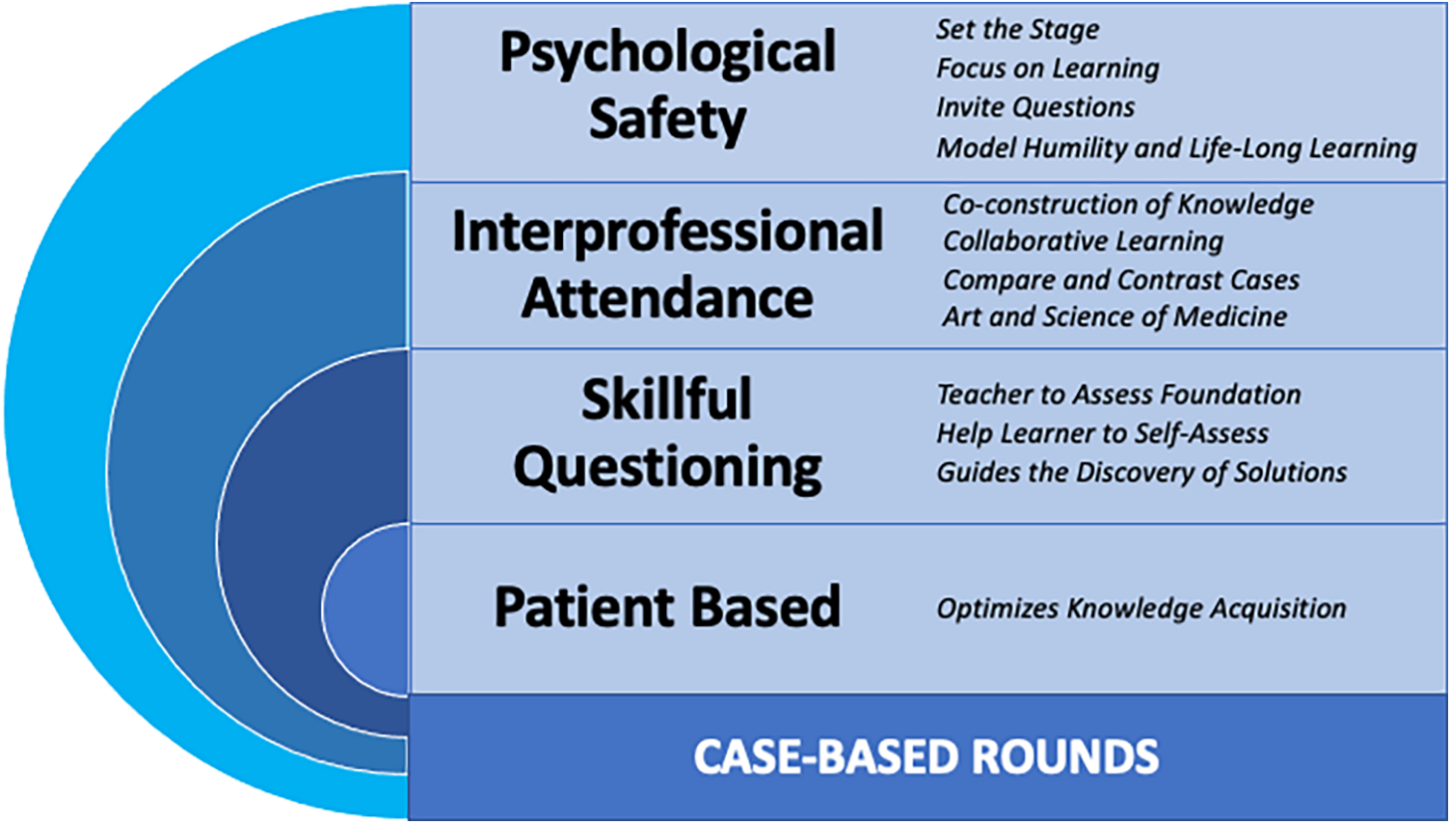
Guiding principles for facilitation of case-based rounds based on the underpinning educational theories. Figure created by authors.

The strengths of case-based rounds can transform into threats if not used properly. Fortunately, these risks can be mitigated and the learning potential from this activity maximized by ensuring the psychological safety of all those involved (19). Psychological safety has been described as “a perception that the environment is safe for the team members to express concern, ask questions, acknowledge a mistake without the fear of humiliation, retaliation, blame or being ignored” (20). In medical education, psychological safety permits the learners to be present and take full advantage of the learning session (19). The following three principles give a rough guideline for the educators to ensure an optimal learning environment (20). First, setting the stage about the goals that the case-based rounds are to accomplish, and their structure, will reduce participants' anxiety. Second, ensure that the focus remains on learning. Third, invite questions and answers, model humility and life-long learning This allows more junior learners to do the same. Reframing gaps as learning opportunities allows participants to thrive. We summarize these important elements in Figure 2.

Another potential threat is inadvertently putting the focus on rare cases and individual clinician decisions or promote defensive medicine (21). The point of these rounds in our institution is to explore the case in detail and compare and contrast with other cases so a better understanding and management, informed by collective experience, may occur. The focus is on key cases that add to the knowledge of intensivists. While the factors that push someone to practice “defensive medicine” are multiple, education seems to be a factor that promotes appropriate management (22).

Having steered the “Morning Rounds” for 40 years and observing that it is the most highly evaluated educational activity, we sought to dissect some of the features that make these rounds successful and look at them from the lens of educational theories. Through this exercise we hope to assist other educational programs who want to establish, modify, or maintain a similar teaching platform to ours. These rounds allow participants to continue to explore important clinical topics raised at the bedside and can facilitate more in-depth learning and support of our learners' journey to clinical excellence. Being a witness and mentor to that journey is highly fulfilling to physician teachers. After all, William Osler reportedly asked for his epitaph to be “he taught medical students in wards”, something that he considered his most useful work.

## Data Availability

The raw data supporting the conclusions of this article will be made available by the authors, without undue reservation.
